# Community- and facility-based HIV testing interventions in northern Tanzania: Midterm results of Test & Treat Project

**DOI:** 10.1371/journal.pone.0266870

**Published:** 2022-04-12

**Authors:** Giulia Martelli, Lukas Van Duffel, Edith Cosmas Kwezi, Francesco Cavallin, Idd Amiri Salehe, Giovanni F. Torelli, Giovanni Putoto, Sabine Hermans, Tobias F. Rinke de Wit, Anton Pozniak

**Affiliations:** 1 Doctors with Africa CUAMM, Shinyanga, Tanzania; 2 Independent statistician, Solagna, Italy; 3 Doctors with Africa CUAMM, Dar es Salaam, Tanzania; 4 Doctors with Africa CUAMM, Padua, Italy; 5 Department of Global Health, Amsterdam Institute for Global Health and Development, Amsterdam UMC, University of Amsterdam, Amsterdam, The Netherlands; 6 HIV and Sexual Health Clinic, Chelsea and Westminster Hospital NHS Foundation Trust London, London, United Kingdom; Hospital Barros Luco, CHILE

## Abstract

Test & Treat Project offers universal HIV testing and access to antiretroviral treatment in Northern Tanzania. The current cross-sectional study provides midterm results on HIV testing and counseling activities through community outreaches and facility-based services. A total 255,329 HIV tests were performed: 198,451 (77.7%) during testing campaigns in the villages, 12,592 (4.9%) during special events outreach and 44,286 (17.4%) in the health facilities. Females represented 53.8% (23,809) among those tested in the health facilities, while males were the majority in the community (54.4%, 114,835). Over one third of tests (n = 104,605, 41%) were performed among first-time testers. The overall HIV positivity rate was 1.2%, ranging from 0.7% in the community to 3.8% in the health facilities and decreased over time. Using a multivariable analysis, a positive test result was associated with age ≥ 50 years (PR 1.22, 95% CI 1.11 to 1.34), with female gender (PR 1.61, 95% CI 1.50 to 1.73), being tested in health facilities (PR 5.00, 95% CI 4.65 to 5.36) and for the first time (PR 1.86, 95% CI 1.73 to 2.00). The estimated proportion of PLHIV who knew their status of the project area increased by 28.6% (from 35.7% to 64.3%) and 11.1% (from 57.7% to 68.8%) in the project areas of Shinyanga and Simiyu regions respectively. Reaching the first UNAIDS 90 target by the end of this project seems possible. Future strategies should focus on improving PITC coverage, implementing more targeted testing modalities, together with current universal community-based approach.

## Introduction

The global HIV epidemic, despite progressive decline since the introduction of antiretroviral therapy (ART), still resulted in 1.7 million new infections in 2018, which means that the UNAIDS target of fewer than 500,000 new infections yearly by 2020 will not be met [1]. About 61% of these new infections take place in sub-Saharan Africa (SSA) [1], where awareness of HIV status among people living with HIV (PLHIV) ranges from 60 to 86%, according to Population-HIV-Surveys [2].

Scaling up HIV testing services is essential to reach the UNAIDS 90:90:90 targets [3]. HIV testing services (HTS) can be either conventional facility-based or community-based. Facility-based testing includes all tests performed at health facilities, either requested by the clients (voluntary counselling and testing, VCT) or health care provider-initiated testing and counselling (PITC). Community-based testing includes different modalities: home-, mobile-, venue-, workplace-based or as part of a campaign [4, 5]. Index testing involves tracing of sexual contacts and children of HIV clients who are enrolled in care and can either be performed at facility level or be integrated in community testing. WHO strongly recommends the implementation of community-based testing [6], since it showed higher population coverage and identified PLHIV at earlier disease stage compared to the conventional HTS in facilities [5]. Moreover, community testing may overcome barriers between clients and the health facilities (distances, costs, long waiting times) [5]. Since contexts vary depending on local HIV epidemics and cultural factors, UNAIDS suggests to tailor testing modalities according to specific regional policies and needs [7]. Previous universal test and treat trials showed that intensive community testing campaigns can have a strong impact on reaching the UNAIDS first 90 target. However, in light of the relatively lower yield of this universal approach, it could be questioned whether more targeted community testing strategies would be preferred [8, 9].

In Tanzania an estimated 78% of PLHIV were aware of their status in 2018; while in the same year 72,000 new HIV infections were estimated to have occurred in the country, with an overall prevalence of 5% [1]. In 2019 Tanzanian Government has introduced HIV testing guidelines, recommending PITC for all patients who access health facilities and community testing for those who have limited access to health care [10]. Despite these efforts, still many PLHIV remain undiagnosed: approximately 22% of Tanzanian women and 46% of men reported to have never been tested in their life [11].

Test & Treat Project (T&TP), a five-year programme which started in 2016, aims at implementing and supporting HTS and HIV care in Shinyanga and Simiyu regions in northern Tanzania.

The main objective of this paper is to describe midterm results of T&TP, displaying the different HIV testing modalities, their testing yields and the socio-demographic characteristics of the population reached. Secondary objectives are: i) to investigate socio-demographic factors and testing modalities associated with being tested for the first time and with being HIV positive; ii) to analyze the number of performed tests and the testing yields over time; iii) to estimate the impact of T&TP in contributing to the first 90 in the project catchment areas.

## Methods

### Study design

This is a cross sectional study, providing results of all testing services implemented and supported by T&TP between May 2017 and June 2019.

### Study settings and population

The project supports four health facilities located in four districts: Shinyanga District Council, Shinyanga Municipal Council in Shinyanga region and Bariadi District Council and Itilima in Simiyu region. The estimated HIV prevalence is 5.9% and 3.9% in Shinyanga and Simiyu regions, respectively [12]. All facilities host a Care and Treatment Clinic (CTC) for HIV clients and hold some inpatient care capacity.

The catchment area is mostly rural and has a population of 461,932 inhabitants [13]. The study population includes all subjects who underwent HIV testing during the study period, performed by any of the T&TP supported testing activities.

### Testing activities

T&TP implements community-based testing in the form of universal mobile outreach campaigns and testing during special events, and supports HTS within the supported health facilities. Testing and counseling procedures for all modalities are conducted in accordance with Tanzania National HTS Guidelines [10, 14].

Community-based testing is carried out by testing teams (one per health facility), composed by four trained nurses and a driver, who offer voluntary testing and counseling. The campaigns take place on normal weekdays, systematically visiting all villages of the catchment area on an approximately yearly basis. Special events are integrated in already existing community activities, such as festivities or other health preventive campaigns. The two modalities offer the same service, but the addressed population might be quite different.

After counseling all clients provide their consent and they are individually screened to assess their eligibility for HTS: clients are considered eligible if they were previously never tested for HIV or tested negative more than 3 months before. The testing algorithm is followed using finger-prick whole blood samples for serial rapid tests: SD BIOLINE HIV I/2-3.0 (Standard Diagnostics, Inc., Gyeonggi, Republic of Korea), followed by Uni-Gold HIV (Trinity Biotech Manufacturing Ltd., Bray, Ireland) only when the first one is reactive. The result is considered positive only when both tests are reactive [10, 14]. In case of discordant results between the two assays another health care worker should repeat the tests; and if the results remain discordant, the client is asked to repeat testing (following the same algorithm) after 14 days. Each newly identified positive client is counselled and offered to immediately go to the nearest CTC for enrollment using the project transportation (motorbike or car), or either accompanied later by a community health worker of the village or autonomously with the referral letter.

T&TP supports facility-based testing by appointing a designated and trained health care worker in each of the facilities, who is involved in HTS at all inpatient and outpatient departments.

### Data collection

After each test performed, both for community- and facility-based testing, individual data are entered in the designated government testing registers of the National AIDS Control Program (NACP). Based on these, testing reports are compiled on daily basis for community-based testing, and on monthly basis for facility-based testing. These include the number of tests performed and of positive tests, aggregated by gender and age categories. At the facility, reports contain results of all tests performed, at all departments and by any modality. From these reports, aggregated data were collected in a dedicated project database.

A de-identified, anonymized and aggregated data set file is provided in the (S1 File).

### Data analysis

All data were categorical and were reported as numbers and percentages. HIV testing yields were defined as the number of positive HIV tests divided by the number of tests performed. HIV tests and testing yields were stratified by age, sex, previous testing history (first-time tester or not) and testing modalities.

Associations between categorical variables were assessed using the Chi Square test. Factors associated with being tested for the first time were investigated using a log-link binomial generalized linear model. Factors associated with HIV positivity were investigated using a log-link binomial generalized linear model. Effect sizes were expressed as prevalence ratio (PR) with 95% confidence interval (CI).

Joinpoint regression analysis was performed to explore trends in number of tests and testing yield, and to identify possible points in time with a significant change. The best-fitting model and the number of joinpoints were estimated by means of a Monte Carlo Permutation method. The trends were expressed as monthly percentage change (MPC), which were estimated using log-linear regression models. Special events were excluded from trend analysis due to their episodic occurrence.

All tests were 2-sided and a p-value less than 0.05 was considered statistically significant. Statistical analysis was performed using the Joinpoint Regression Program, version 4.1.1 [15] and R 3.5 [16]. Based on previous reports of the regional HIV prevalence, demographics and testing cascade [12, 13], the contribution of T&TP to the first 90 was estimated. Details of methodology and results are shown in S1 and S2 Tables.

### Ethical considerations

A specific Memorandum of Understanding was signed in 2016 between Doctors with Africa CUAMM and the Regional Medical Officers (RMO) of Shinyanga and Simiyu Regions before starting the implementation of the activities. The study was approved by the Institutional Review Board of National Institute for Medical Research (NIMR) of Tanzania (NIMR/HQ/R.8c/Vol.I/1447). Data clerks employed by the project transferred the data entry from governmental reports to a project electronical database. All managed data were aggregated and, therefore, anonymous. All data were stored in a password-protected server, which only the researchers, the project manager and coordinators had access to. On monthly basis, the testing activities in the community were reported at district level in order to inform the authorities about the results achieved and to avoid overlap with other stakeholders’ projects.

## Results

### Test conducted by modalities and demographics

A total 255,329 HIV tests were performed: 198,451 (77.7%) during testing campaigns, 12,592 (4.9%) during special events and 44,286 (17.4%) in the health facilities. More than one third of the individuals accessing the health facilities for any reason (38.2%, 44,286 out of 115,979) were offered testing and counselling for HIV. Characteristics of tests performed are shown in Tables 1 and 2. Gender distribution varied among testing modalities: females represented 53.8% (23,809) among those who tested in the health facilities, while males were the majority in the community (54.4%, 114,835 among testing campaigns and special events; p<0.0001). Testing in the community involved younger participants (median 15–24 years) compared to health facility-based testing (median 25–49 years; p<0.0001). Comparing the two modalities for community-based testing, the proportion of males was higher in the special events than in the testing campaigns (64.1% versus 53.8%), and the special events attracted less children (2.5% vs 22.6% in the testing campaigns).

**Table 1:** Socio-demographic characteristics of performed tests according to testing sites and modalities.

	Community-based		Facility-based	p-value
	Testing campaign	Special events		
	N (% by column)	N (% by column)	N (% by column)	-
Overall	198,451	12,592	44,286	-
Females	91,684 (46.2)	4,524 (35.9)	23,809 (53.8)	<0.0001
Males	106,767 (53.8)	8,068 (64.1)	20,477 (46.2)	
Age:				<0.0001
≤14 years	44,847 (22.6)	312 (2.5)	6,712 (15.2)	
15–24 years	59,352 (29.9)	4,118 (32.7)	13,335 (30.1)	
25–49 years	77,302 (39.0)	6,829 (54.2)	19,369 (43.7)	
≥ 50 years	16,950 (8.5)	1,333 (10.6)	4,870 (11.0)	
First-time testers	84,034 (42.3)	3,600 (28.6)	16,971 (38.3)	<0.0001
Re-testers	111,417 (57.7)	8,992 (71.4)	27,315 (61.7)	

**Table 2:** Characteristics of HIV tests performed among first-time testers and re-testers.

	All	Re-testers	First-time testers	p-value
	N (% by column)	N (% by column)	N (% by column)	-
Overall	255,329	150,724 (59.0)	104,605 (41.0)	-
Testing campaigns	198,451 (77.7)	114,417 (75.9)	84,034 (80.4)	<0.0001
Special events	12,592 (4.9)	8,992 (6.0)	3,600 (3.4)	
Facility-based	44,286 (17.4)	27,315 (18.1)	16,971 (16.2)	
Females	120,017 (47.0)	70,479 (46.8)	49.538 (47.4)	0.003
Males	135,312 (53.0)	80,245 (53.2)	55,067 (52.6)	
Age:				<0.0001
≤14 years	51,871 (20.3)	6,482 (4.3)	45,389 (43.4)	
15–24 years	76,805 (30.1)	46,062 (30.6)	30,743 (29.4)	
25–49 years	103,500 (40.5)	81,359 (54.0)	22,141 (21.2)	
≥ 50 years	23,513 (9.1)	16,821 (11.1)	6,332 (6.0)	

### First-time testers and associated factors

Among the testers, 41.0% (104,605) declared it was their first in a life-time test, with a higher proportion among those younger than 25 years (59.2% versus 22.4%, p<0.0001). At multivariable analysis, first-time tests were more likely to occur during community-based testing (PR 1.02; 95% CI 1.01 to 1.04), among males (PR 1.05, 95% CI 1.04 to 1.07) and participants aged < 25 (≤14 years: PR 7.31, 95% CI 7.19 to 7.43; 15–24 years: PR 2.13 95% CI 2.10 to 2.17) and ≥ 50 years (PR 1.33, 95% CI 1.29 to 1.37) (S3 Table).

### Testing yields, associated factors and trends over time

Overall, the HIV positivity rate was 1.2% (3,114 positives out of 255,329 tests performed), ranging from 0.7% in the community (0.6% in testing campaign and 1.2% in special events) to 3.8% in the health facilities. Summary of testing yields is shown in Tables 3 and 4.

**Table 3:** Positivity rates among first-time testers and re-testers.

	Positivity rate among all testers	Positivity rate among re- testers	Positivity rate among first-time testers
	N (% on all testers)	N (% on re-testers)	N (% on first-time testers)
Overall	3,114 (1.2)	1,781 (1.2)	1,333 (1.3)
Testing campaigns	1,267 (0.6)	791 (0.7)	476 (0.6)
Special events	156 (1.2)	121 (1.3)	35 (1.0)
Health facility-based	1,691 (3.8)	869 (3.2)	822 (4.8)
Females	1,871 (1.6)	1,105 (1.6)	766 (1.5)
Males	1,243 (0.9)	676 (0.8)	567 (1.0)
Age:			
≤14 years	147 (0.3)	19 (0.3)	128 (0.3)
15–24 years	520 (0.7)	306 (0.7)	214 (0.7)
25–49 years	1,888 (1.8)	1,175 (1.4)	713 (3.2)
≥ 50 years	559 (2.4)	281 (1.7)	278 (4.4)

**Table 4:** Positivity rates stratified by testing modalities, gender and age.

	HIV-positives / tested (%)					
	Campaigns		Special events		Health facilities	
Age classes	Females	Males	Females	Males	Females	Males
≤14 years	25/23,053 (0.1)	19/21,794 (0.1)	0/180 (0.0)	1/132 (0.8)	51/3,483 (1.5)	51/3,229 (1.6)
15–24 years	174/26,182 (0.7)	65/33,179 (0.2)	20/1,434 (1.4)	7/2,684. (0.3)	203/7,918 (2.6)	51/5,417 (0.9)
25–49 years	442/34,900 (1.3)	303/42,402 (0.7)	52/2,423 (2.1)	53/4,406 (1.2)	595/10,228 (5.8)	443/9,141 (4.8)
≥ 50 years	144/7,549 (1.9)	95/9,401 (1.0)	15/487 (3.1)	8/846 (0.9)	150/2,180 (6.9)	147/2,690 (5.5)

HIV-positivity rate was higher in females compared to males both in the community (0.9% vs. 0.5%, p<0.0001) and in the health facilities (4.2% vs. 3.4%, p<0.0001) and varied among age categories (p<0.0001), with highest rate among those ≥ 50 years both in the community (1.4%) and in the health facilities (6.1%).

At multivariable analysis, being HIV positive was associated with age ≥ 50 years (PR 1.22, 95% CI 1.11 to 1.34), being female (PR 1.61, 95% CI 1.50 to 1.73), being tested for the first time (PR 1.86, 95% CI 1.73 to 2.00) and being tested in health facilities (PR 5.00, 95% CI 4.65 to 5.36). On the other hand, lower HIV positivity rate was associated with age ≤ 24 (age ≤14 years: PR 0.12, 95% CI 0.10 to 0.14; age in 15–24 years: PR 0.33, 95% CI 0.30 to 0.37) (S4 Table). Comparing the two community modalities, individuals tested during special events were more likely to test HIV positive compared to those accessing the campaigns (PR 1.67, 95% CI 1.41–1.97).

**Fig 1:**
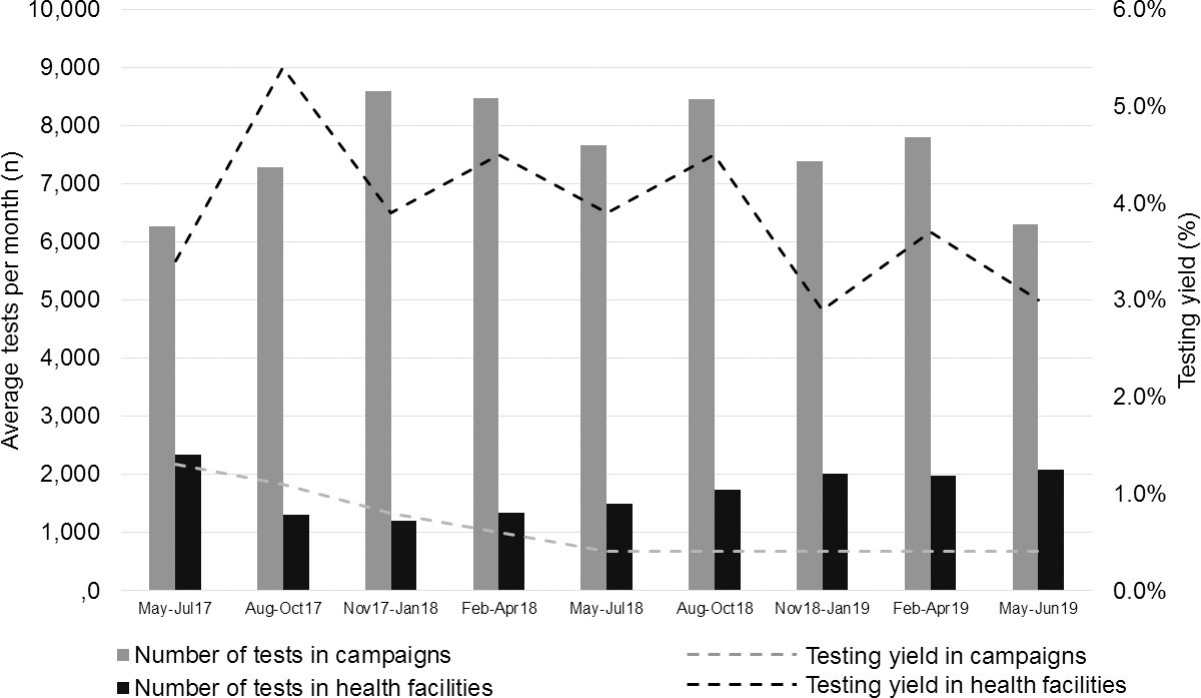
Number of tests performed and testing yields of HIV positives over time among the testing campaigns and within the health facilities. Number of tests is shown as average number per month in y-axis due the duration of the last sub-period (May-June 2019; 2 months) with respect to the duration of the other sub-periods (3 months). Data from special events were excluded from this model due to their episodic occurrence.

The proportion of clients found HIV positive decreased over time from 2.0% in May 2017 to 1.0% in June 2019, with different slopes among the testing campaigns and the health facilities, as displayed in Fig 1. Estimated testing yield decreased in the first 14 months (MPC -9.69%, p<0.0001) then levelled (MPC –1.94%, p = 0.33) among testing campaigns, while it decreased over study period (MPC -1.26%, p = 0.04) within the health facilities, but with a more irregular curve.

### Impact on the first 90

The estimation shows that during the study period, the proportion of PLHIV who knew their status increased by 28.6% (from 35.7% to 64.3%) and 11.1% (from 57.7% to 68.8%), in the project areas of Shinyanga and Simiyu regions respectively (see S1 Table).

The S2 Table shows that the estimated proportion of PLHIV in the area who did not know their status before the project started was 1.6%.

## Discussion

Over a two-year period, T&TP has performed more than a quarter million HIV tests, identifying 3,114 positives. Furthermore, 41% of the tests were conducted among individuals who reported to have never been tested before in their lifetime. The positivity rate was slightly lower than expected and decreased over time. We estimated that the project made a significant contribution towards UNAIDS first 90, aiming to reach the target by the end of the project.

Among the 255,329 HIV tests performed in the first phase of T&TP, the majority took place at community level (82.6%, 211,043), where a higher proportion of males and young adults were observed. This is in line with other reports in SSA [4, 17, 18], with the exception of home testing (not applied in our testing activities), which is more accepted among women [5, 19]. Males might be reluctant to access health facilities for many reasons, among which perceived lack of confidentiality in medical structures, fear of stigma and a feeling of masculinity translated as being strong and healthy and therefore avoiding to seek care [17]. On the other hand, women might feel more comfortable accessing facility-based HTS, given the major role played by routine HIV testing at antenatal clinics [11, 20]. Additionally, women who recently underwent testing at the health facility might be less inclined to participate at community campaigns.

PITC remains probably the greatest and most cost-effective contributor to the first 90 [3, 4, 21]; aiming ideally at 100% PITC coverage should therefore be a primary goal [10, 22]. However, in practice, coverage is often low as a result of multiple challenges, among which understaffing, lack of trained counselors, overcrowding of wards and clinics, and reagents stockouts [5]. Despite the provided support, the coverage of 38.1% at the T&TP facilities is lower than previously described in other research settings [23].

Across the different modalities, 41% of the tests were performed among participants who reported to be first-time testers, which is similar to observations made in a national survey in Kenya (43.1% of 16–64 years participants) [20], and a home-based testing intervention in Zambia (35.2%) [24]. This information could be obtained only by client’s self-reporting and is therefore difficult to verify. Nevertheless, despite universal access to HTS being already recommended in Tanzania since several years [14], the high proportion of first-time testers indicates that this service continues to reduce a significant gap in people’s knowledge of their HIV status.

Among our population the first-time testers were more likely to be males, below 25 years and to be tested in the community. These findings were also reflected in a large Tanzanian survey, where, among others, men, young adults (18–24) and people living in rural areas more often reported to never have been tested for HIV [11]. The higher proportion of first-time testers in the community-based approach might be explained by the fact that community HTS brings the service closer to healthier individuals, who otherwise might not have reasons to access conventional facility-based HTS. That is also why community HTS identifies HIV individuals at an earlier, mostly asymptomatic stage [18, 25]. During interviews with clients who participated to the testing campaign, it was indeed confirmed that the possibility to test nearby home was among the reasons to get tested (Josien de Klerk, personal communication).

The overall HIV positivity rate was 1.2%, which is slightly lower than the estimated proportion of PLHIV in the area who did not know their status before the project started (1.6%, see S2 Table). However, to note, an unknown number of PLHIV who were tested positive in our HTS might have already known their status, thus falsely increasing the positivity rate.

The yield was considerably higher in the health facilities (3.8%) than in the community-based approaches (0.6%). This contrast has been extensively described previously [4, 25, 26] and is explained by the higher proportion of symptomatic individuals being tested at facility level [25].

The relatively lower yields observed in the community might be related to many factors: i) universal community testing might not be able to target key populations, due to stigma; ii) some HIV negative individuals are likely to be retested over time; iii) the majority of individuals who are HIV positive in the community might be already identified and linked to care, due to interventions of other stakeholders; iv) HIV prevalence in these specific, mostly rural geographic areas might be lower than in previous regional reports, in which the urban positivity rates might have skewed the overall estimation.

As compared to the mobile outreach campaigns, the special events showed a higher positivity rate, as well as higher participation of men. Similar observations were made in a testing intervention in Northern Tanzania where event-based testing was compared with home-based universal testing [4].

Those who tested for the first time showed significantly higher yields compared to re-testers: Sharma et al reported this trend, defining the first-time testers among the target group which mostly benefit from HTS implementation [5] due to their lack of perception of risks [18].

Higher positivity rates found among females and individuals above 25 years old reflect the characteristics of HIV epidemics in SSA [1, 19]. However, we found the highest proportion of HIV positivity among the age category above 50 years, which slightly diverges from the country scenario described by the Population-based HIV Impact Assessment (PHIA), where prevalence among this category is lower than the age group 35–50 [12]. Other authors described higher prevalence among older adults in Tanzania [27, 28], suggesting that, against the perception that HIV/AIDS is mainly a disease of the young, interventions targeting older populations might be implemented in parallel with other strategies.

Finally, the positivity rate significantly decreased over the analyzed time frame and this was more evident among the tests performed in the community through testing campaigns. Despite the relatively short timeframe, this could reflect a general reduction of HIV incidence in Tanzania, as described for SSA [1]. On the other hand, as the project revisited each vicinity over time, individuals who are more concerned about their health status could have been more likely to get retested; leading to an overrepresentation of what could be considered a lower risk population. Social research showed that concerns about one’s health status were indeed among the reasons for retesting. However, some re-testers were driven by mistrust of the sexual partner(s) and can therefore hardly be considered to be part of a lower-risk population (Josien de Klerk, personal communication).

The relatively low yield and perceived higher costs could be arguments to shift from universal community testing to more targeted approaches, such as index testing, self-testing and hot spot testing [22]. Also, a focus on increasing ART coverage for those populations with higher transmission risk could have more impact on incidence [9].

However, while implementing these focused strategies, there are many arguments in favor of continuing universal community-based testing modalities. First of all, targeted strategies are more laborious and time consuming, therefore, higher yield does not necessarily result in a higher absolute number of positives found [3, 8]. Secondly, the community testing activities, more than merely finding new positives, promote an extensive process of sensitization and counselling at community level, the value of which cannot be underestimated [19]. Furthermore, social researchers of T&TP reported that the community-based universal testing offers the ability to keep monitoring one’s health status, to reassure oneself when felt at risk, or even to confirm a previously positive but doubtfully perceived test (Josien de Klerk, personal communication). Finally, in the effort to reach universal health coverage, outreach campaigns are an opportunity to offer integrated prevention and screening services for many diseases [8, 29].

This work has some strengths: i) its specificity to the context of Shinyanga and Simiyu regions, described among those with highest HIV prevalence in the country [12], ii) a very large sample size, which allows to draw important observations, iii) the comparison between community versus facility-based HTS.

The main limitation of this study was the use of aggregated data, which restricted the statistical analysis, precludes the identification of individuals who got retested over time and did not allow for linkage to individual clinical patient data.

Secondly, the proportion of newly identified cases was not measured since accurate data on PLHIV who already knew their status was lacking.

Thirdly, data on population testing coverage in the catchment areas of the outreach campaign are not available.

This midterm analysis was a useful tool for reviewing the implementation of HTS of T&TP. Its approach managed to reach and sensitize a large population, among which substantial proportions of men, young individuals and first-time testers. In order to be able to reach the first 90 by the end of the project, future strategies should focus on improving PITC coverage, continuing universal community-based testing, while expanding and optimising more targeted modalities, such as special events, key population approaches and index testing.

## Supporting information

S1 File Dataset. (XLSX)

S1 Table Calculation of contribution of first 90 in the catchment area of T&TP [30]. (DOCX)

S2 Table Calculation of expected proportion of PLHIV still to be identified in the catchment area of T&TP. (DOCX)

S3 Table Multivariable analysis of factors associated with first-time test. (DOCX)

S4 Table Multivariable analysis of factors associated with HIV positivity. (DOCX)
