# Supplementary material for: Community- and facility-based HIV testing interventions in northern Tanzania: Midterm results of Test & Treat Project
Source: PLoS One. 2022 Apr 12;17(4):e0266870. doi: 10.1371/journal.pone.0266870 (PMC9004748; doi:10.1371/journal.pone.0266870)
Supplement: S1 Table — (DOCX) [file pone.0266870.s002.docx]

## **S1 Table**. Calculation of contribution of first 90 in the catchment area of T&TP

| 1. Population of T&TP coverage area (13,30)   185,066 (Shinyanga) + 276,866 (Simiyu) = 461,932 in 2012  214,768 (Shinyanga) + 320,964 (Simiyu) = 535,732 estimated in 2017 (3% yearly growth rate) |
| --- |
| 1. Estimated regional prevalence (12)   5.9% Shinyanga and 3.9% Simiyu (among adult population)  0.6% Shinyanga and 0.9% in Simiyu (among children < 15 years) |
| 1. Estimated number of PLHIV in the coverage area (12,31)   214,768 * 55.2% (proportion of adult population in the country) * 5.9% = 6,995 PLHIV adult in Shinyanga  214,768 * 44.8% (proportion of children < 15 years in the country) * 0.6% = 711 PLHIV children in Shinyanga  6,995 + 711 = 7,706 PLHIV in Shinyanga  320,964 * 55.2% (proportion of adult population in the country) * 3.9% = 6,910 PLHIV adult in Simiyu  320,964 * 44.8% (proportion of children < 15 years in the country) * 0.9% = 1,294 PLHIV children in Simiyu  6,910 + 1,294 = 8,204 PLHIV in Simiyu |
| 1. Estimated proportion of PLHIV who know their status in the coverage area (12)   35.7% (Shinyanga) and 57.5% (Simiyu) |
| 1. Estimated number of PLHIV who know their status in the coverage area   2,751 (Shinyanga) + 4,717 (Simiyu) = 7,468 |
| 1. Number positive tests performed in T&TP testing activities   2206 (Shinyanga) + 908 (Simiyu) = 3,114 |
| 1. Contribution to the first 90 in the coverage area   Shinyanga = 28.6%  Simiyu = 11,1% |

This calculation includes some theoretical assumptions due to limited availability of detailed local data and for reasons of simplification:

- The population estimate of the coverage area dates back to 2012, while testing activities started in 2017. Considering the general demographic trends in Tanzania, an estimation of the population size, based on country annual population growth rate (30), was performed;
- The proportion of children and adults among the population of entire Tanzania was assumed to reflect the one of this specific area;
- HIV prevalence in the project’s catchment area was assumed to reflect the regional one (line 3). As the catchment area is mostly peri-urban and rural, differences with the urban epidemic could have altered this estimation;
- Similarly, estimation of number of PLHIV aware of their status in the project’s catchment was assumed to reflect the regional one (line 5);
- The number of individuals who tested positive already aware of their status were not removed from the calculation, due to lack of reliable data.
- Some of the testing campaigns and special events involved people coming from outside the coverage area of the project, hence the estimated population might have been larger and therefore our contribution lower.
