# Supplementary material for: Community- and facility-based HIV testing interventions in northern Tanzania: Midterm results of Test & Treat Project
Source: PLoS One. 2022 Apr 12;17(4):e0266870. doi: 10.1371/journal.pone.0266870 (PMC9004748; doi:10.1371/journal.pone.0266870)
Supplement: S2 Table — (DOCX) [file pone.0266870.s003.docx]

## **S2 Table**. Calculation of expected proportion of PLHIV still to be identified in the catchment area of T&TP

| 1. Population of T&TP coverage area (13,30)   185,066 (Shinyanga) + 276,866 (Simiyu) = 461,932 in 2012  214,768 (Shinyanga) + 320,964 (Simiyu) = 535,732 estimated in 2017 (3% yearly growth rate) |
| --- |
| 1. Estimated regional prevalence (12)   5.9% Shinyanga and 3.9% Simiyu (among adult population)  0.6% Shinyanga and 0.9% in Simiyu (among children < 15 years) |
| 1. Estimated number of PLHIV in the coverage area (12,31)   214,768 * 55.2% (proportion of adult population in the country) * 5.9% = 6,995 PLHIV adult in Shinyanga  214,768 * 44.8% (proportion of children < 15 years in the country) * 0.6% = 711 PLHIV children in Shinyanga  6,995 + 711 = 7,706 PLHIV in Shinyanga  320,964 * 55.2% (proportion of adult population in the country) * 3.9% = 6,910 PLHIV adult in Simiyu  320,964 * 44.8% (proportion of children < 15 years in the country) * 0.9% = 1,294 PLHIV children in Simiyu  6,910 + 1,294 = 8,204 PLHIV in Simiyu |
| 1. Estimated proportion of PLHIV who know their status in the coverage area (12)   35.7% (Shinyanga) and 57.5% (Simiyu) |
| 1. Estimated number of PLHIV who know their status in the coverage area   2,751 (Shinyanga) + 4,717 (Simiyu) = 7,468 |
| 1. Estimated number of PLHIV who don’t know their status in the coverage area   4,955 (Shinyanga) + 3,487 (Simiyu) = 8,442 |
| 1. Estimated proportion of PLHIV still to be identified among the population of the catchment area of T&TP   Shinyanga = 2.3%  Simiyu = 1.1%  Overall = 1.6% |
