# Supplementary material for: Community- and facility-based HIV testing interventions in northern Tanzania: Midterm results of Test & Treat Project
Source: PLoS One. 2022 Apr 12;17(4):e0266870. doi: 10.1371/journal.pone.0266870 (PMC9004748; doi:10.1371/journal.pone.0266870)
Supplement: S3 Table — (DOCX) [file pone.0266870.s004.docx]

## **S3 Table**. Multivariable analysis of factors associated with first-time test

|  | Prevalence ratio (95% confidence interval) | p-value |
| --- | --- | --- |
| Health facility-based  Community-based | Reference  1.02 (1.01-1.04) | -  <0.0001 |
| Females  Males | Reference  1.05 (1.04 to 1.07) | -  <0.0001 |
| Age:  ≤14 years  15-24 years  25-49 years  ≥ 50 years | 7.31 (7.19 to 7.43)  2.13 (2.10 to 2.17)  Reference  1.33 (1.29 to 1.37) | <0.0001  <0.0001  -  <0.0001 |
