# Supplementary material for: Community- and facility-based HIV testing interventions in northern Tanzania: Midterm results of Test & Treat Project
Source: PLoS One. 2022 Apr 12;17(4):e0266870. doi: 10.1371/journal.pone.0266870 (PMC9004748; doi:10.1371/journal.pone.0266870)
Supplement: S4 Table — (DOCX) [file pone.0266870.s005.docx]

## **S4 Table**. Multivariable analysis of factors associated with HIV positivity

|  | Prevalence ratio (95% confidence interval) | p-value |
| --- | --- | --- |
| Already tested  First-time tested | Reference  1.86 (1.73 to 2.00) | -  <0.0001 |
| Community-based  Health facility-based | Reference  5.00 (4.65 to 5.36) | -  <0.0001 |
| Females  Males | 1.61 (1.50 to 1.73)  Reference | <0.0001  - |
| Age:  ≤14 years  15-24 years  25-49 years  ≥ 50 years | 0.12 (0.10 to 0.14)  0.33 (0.30 to 0.37)  Reference  1.22 (1.11 to 1.34) | <0.0001  <0.0001  -  <0.0001 |
